# The structure and *in vitro* starch digestibility of wheat starch-glycerol monopalmitin complexes: the effect of heating conditions and water content

**DOI:** 10.3389/fnut.2023.1288067

**Published:** 2023-11-18

**Authors:** Fengping Jiao, Zesong Liu, Jing Ni, Jinglin Yu, Shujun Wang, Xia Liu

**Affiliations:** ^1^School of Public Health, Shandong First Medical University and Shandong Academy of Medical Sciences, Ji’nan, Shandong, China; ^2^State Key Laboratory of Food Nutrition and Safety, Tianjin University of Science and Technology, Tianjin, China; ^3^School of Food Engineering and Biotechnology, Tianjin University of Science and Technology, Tianjin, China

**Keywords:** heat condition, water content, wheat starch-glycerol monopalmitin complexes, structure, *in vitro* starch digestibility

## Abstract

The effects of the heating conditions and water content on the structure and digestibility of wheat starch (WS)-glycerol monopalmitin (GMP) complexes were investigated. The results showed that the higher water content and the heating conditions of 90°C for 60 min after 100°C for 10 min favor the formation of more WS-GMP complexes with the greater short-range order, although the thermal transition temperatures of GWS-GMP-100 complexes are not significantly affected by the water content. Only the type I complexes were formed under the heating conditions of 90°C for 60 min. The heating conditions of 90°C for 60 min after 100°C for 10 min facilitates the formation of type II complexes, and the amounts of type II complexes increased with increasing water content. The digestion rates of WS-GMP complexes decreased slightly with increasing water content, and the extent of starch amylolysis of WS-GMP complexes significantly decreased after heating further at 90°C compared with that only heating at 100°C. The digestibility of complexes is mainly related to structural order rather than the number of complexes. This study is helpful to further understand starch-lipid complexes by showing that heating conditions and water content influence the formation of WS-GMP complexes.

## 1 Introduction

Starch is the main component of green plants and provides most of the energy needed for people’s daily life. It is composed of two glucose polymers: amylopectin (AP), which has highly branched molecules, and amylose (AM), a linear polymer with a few branches (1–3). Lipids are added to food to enhance the flavor, mouth-feel and nutritive value during processing (4). Given the structural characteristics of AM with helical cavity, the hydrocarbon portion of guest molecules [e.g., fatty acids (FAs), monoglycerides (MGs)] can insert into this hydrophobic cavity to form AM-lipids complexes through hydrogen bonds and hydrophobic interaction (5–10). However, only a few AP can form complexes due to the larger steric hindrance of AP with more and shorter branch chain (1, 4, 11). During food processing, the interactions of AM and lipids can affect quality of finished food, such as texture and digestibility (12). Therefore, starch-lipids complexes have been a topic of wide concern.

There are many factors that can affect the formation of starch-lipid complexes, such as the type of starch and lipid, carbon chain lengths and the unsaturation degree of the lipids, amylose content and the polymerization degree of starch, reaction conditions, and methods of preparation (13, 14). Hydrothermal treatment is the most commonly used method to prepare starch-lipid complexes because most starchy foods need to be cooked with a certain amount of water (15). During this process, heating conditions (heating time and temperature) and moisture content can affect the formation of starch-lipid complexes. As increased water content, the amount of type II starch-lipid complexes with more ordered structure increased (16). However, the opposite results were observed in almond flour during extrusion cooking and rice with parboiling (17, 18). The difference in these results suggests that how does the water content affect complexes formation has not been elucidated clearly.

Glycerol monopalmitin (GMP), a linear molecule synthesized by esterification of palmitic acid and glycerol. In previous studies on the effects of temperature treatment (19), hydrophilic groups (20), different types of lipids (21), and different chain length of monoglycerides (22) on complex formation, the results showed that GMP can form complexes with starch and it has a greater emulsifying action and compounding ability with AM (21). Wheat starch is the main ingredient in staple of the daily diet (such as bread, noodles, biscuits, etc.), and GMP is added as an emulsifier in the processing of these foods to improve the food quality. The addition of GMP can interact with WS, and factors such as moisture content and heating conditions during processing can affect this interaction, which in turn affects the quality and functional properties of food. Therefore, GMP and WS were selected as representative of lipid and starch, respectively. In this study, we prepared wheat starch (WS)-glycerol monopalmitin (GMP) complexes through the control of water content under two different heating conditions. In one condition, WS and GMP were heated at 100°C for 10 min, and in the other condition, they were heated at 100°C for 10 min followed by 90°C for 60 min. We speculated that heating conditions and water content may influence the interaction and complexes formation between WS and GMP.

Hence, the effects of the heating conditions and water content on the formation, structure, and digestibility of WS-GMP complexes were investigated in this study. A better understanding of this topic will be helpful to understand deeply on the effect of the heating conditions and water content on the formation of WS-GMP complexes and modulate the functionality and nutrition of starchy food.

## 2 Materials and methods

### 2.1 Materials

Wheat starch (9.0% moisture content, 27.5% amylose content, and 0.18% lipid content), glycerol monopalmitin (GMP, C16:0) and α-amylase (Sigma, EC 3.2.1.1, type VI-B from porcine pancreas, 13 units/mg) were obtained from Sigma Chemical Co. (St. Louis, MO, USA). The Glucose Oxidase/Peroxidase Kit (GOPOD format) and Aspergillus niger amyloglucosidase (3260 units/mL) were purchased from Megazyme International Ireland, Ltd. (Bray Co., Wicklow, Ireland). All other chemical reagents were of analytical grade.

### 2.2 Preparation of starch-lipid complexes

Native wheat starch (WS, 2.0 g, wet weigh basis) was weighed and added distilled water to obtain starch suspensions with 50, 60, 70, 80, and 90% moisture contents (w/w, wet starch basis). Subsequently, 100 mg GMP was added and mixed thoroughly under magnetic stirring. And the partial mixtures were heated at 100°C for 10 min (designated WS (m%)-GMP-100), whereas other the mixtures of WS and GMP were firstly heated at 100°C for 10 min and then heated at 90°C for 60 min (WS (*n*%)-GMP-100-90). All above obtained samples were freeze-dried and ground into powders.

### 2.3 Differential scanning calorimetry (DSC)

Thermal properties of the WS-GMP complexes were examined using a differential scanning calorimeter (200 F3, Netzsch, Germany) according to the method described in the previous study with minor modification (23). Differently, samples were heated from 20 to 130°C at a heating rate of 10°C/min. The onset (T_*o*_), peak (T_*p*_), conclusion (T_*c*_) temperatures, and gelatinization enthalpy change (ΔH) were obtained through data recording software.

### 2.4 X-ray diffraction analysis (XRD)

The crystalline structure of WS-GMP complexes was determined using a Bruker D8 Advance X-ray diffractometer (Bruker, Germany) operating at 40 kV and 40 mA. Samples were equilibrated over a saturated NaCl solution for 1 week before measurement. XRD patterns were obtained from 5 to 35° (2θ) at a rate of 2°/min and a step size of 0.06° (24).

### 2.5 Laser confocal micro-Raman spectroscopy

Renishaw Invia Raman microscope system (Renishaw, Gloucestershire, UK) equipped with a Leica microscope (Leica Biosystems, Wetzlar, Germany) was used to collect spectra in the range of 3200–100 cm^–1^ with a resolution of approximately 7 cm^–1^. The full width at half maximum of the band at 480 cm^–1^ (FWHM_480_) was calculated to characterize the short-range molecular order of WS-GMP complexes by the WIRE 2.0 software (21).

### 2.6 *In vitro* enzymatic digestion

*In vitro* enzymatic digestibility of WS-GMP complexes was determined according to the procedure described elsewhere (25). Differently, the WS-GMP complexes were incubated 4 h and determined the glucose content. The digestion curve was drawn with the release of glucose as a function of digestion time. The first-order rate coefficient (k) was fitted, and the content of rapidly digested starch (RDS, digested within 20 min), slowly digested starch (SDS, digested between 20 and 120 min) and resistant starch (RS, undigested after 120 min) were calculated based on the digestion curve.

### 2.7 Statistical analysis

In addition to XRD, all characterizations performed triplicate measurements, and the results in tables are reported as the mean values and standard deviations (SDs) obtained by the SPSS 17.0 Statistical Software Program (SPSS, Inc. Chicago, IL, USA). Pearson’s correlation analysis was also conducted by the SPSS 17.0 statistical software.

## 3 Results and discussion

### 3.1 Thermal properties of complex samples

**FIGURE 1:**
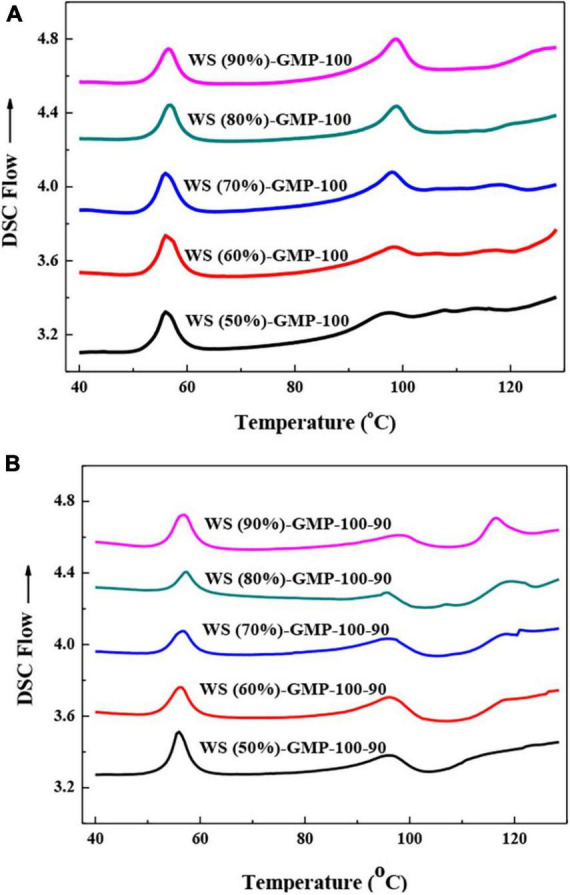
Differential scanning calorimetry (DSC) curves of WS-GMP complexes. **(A)** WS-GMP complexes were prepared at 100°C for 10 min. **(B)** WS-GMP complexes were prepared at 100°C for 10 min followed by complexing at 90°C for 60 min. WS (no/o), wheat starch with no/o water content; GMP, glycerol monopalmitin.

Figure 1 shows the DSC thermograms of WS-GMP complexes. All WS-GMP-100 complex samples exhibit two endothermic peaks. The peak at 50–65°C attributed to the melting of uncomplexed GMP and the second at 85–103°C is the typical thermal transitions peak of the WS-GMP-100 complexes. Previous studies have reported that starch-lipid complexes exist in two distinct crystalline forms depending on crystallization temperature. Type I complexes consisting of non-crystalline helical segments and crystalline V-type packing has a lower thermal transition temperature between 95 and 105°C, and type II with distinct crystalline structures and amorphous regions has a higher melting temperature (14, 26). Different from WS-GMP-100 complex samples, WS-GMP-100-90 complex samples showed different thermal transitions according to the water content. WS (50%)-GMP-100-90 showed two endothermic transitions, which respectively represent the melting of uncomplexed GMP (around 53–60°C) and type I complexes (around 90–103°C). However, three endothermic transitions, related to the melting of uncomplexed GMP, type I complexes and type II complexes (around 110–120°C) were observed for other WS-GMP-100-90 complexes. These results suggest that the lower water content was more readily to form less stable type I WS-GMP-100-90 complexes, whereas the higher water content tended to form type I and type II WS-GMP-100-90 complexes. No type II complexes were found in WS-GMP-100 complexes even under conditions of higher moisture content, which may be due to the fact that the shorter heating time and the heating temperature of 100°C do not allow stable type II complexes to form.

The thermal transition temperatures (T_*o*_, T_*p*_, T_*c*_) and enthalpy change (ΔH) of WS-GMP complexes are presented in Table 1. The ΔH value can reflect the amounts of complexes (27). The total enthalpy change (ΔH_*t*_) of WS-GMP complexes prepared under two heating conditions increased with the increasing water content, for example, from 2.6 J/g for WS (50%)-GMP-100 to 5.3 J/g for WS (90%)-GMP-100, suggests that the complex formation during heating can be controlled by the water content, and the higher water content was more favorable for the formation of WS-GMP complexes. A previous study found that as the water content increased from 6 to 40%, the amount of complex formation increased, and as the water content further increased from 40 to 60%, the amount of complex decreased (28). The different results with this study may be related to processing methods and lipid types. The results of this study are due to that the better starch chain dispersibility of the WS with higher water content makes the GMP insert easily into the hydrophobic cavity of AM to form WS-GMP complexes (29). But thermal transition temperatures of WS-GMP-100 complexes seemed to be little affected by the water content. WS-GMP-100-90 complexes have higher ΔH_*t*_ than the corresponding WS-GMP-100 complexes, indicating that the complexes were formed further after heating further at 90°C for 60 min. A previous study found that the heating conditions of 90°C for longer exhibited a decrease in the amount for type I polymorphs and an increase in type II polymorphs with increased thermal stability (10). The results of this study are attributed to the longer heating time and the slow nucleation of the complexes due to the further heating treatment at 90°C. More than that, the amounts of type II complexes were gradually increased with increasing water content for WS-GMP-100-90 complexes. More specifically, WS (50%)-GMP-100-90 had a ΔH_1_ value of 3.3 J/g from only type I complexes. As the water content increased, the ΔH_1_ values of type I complexes decreased from 4.6 to 3.3 J/g and the ΔH_2_ values of type II complexes increased from 0.9 to 3.7 J/g. At the condition of lower water content, dispersed starch chains in gelatinized starch interact strongly with each other, resulting in less availability of these chains for lipids to complex. Due to the limited space in the condition of the lower water content, the formed complexes were not ready to arrange into stable V-type crystalline structure. However, starch chains with the condition of higher water content are more dispersed and available for the lipids to complex, and the formed complexes are arranged more easily into stable type II complexes.

**TABLE 1:** Thermal parameters of WS-GMP complexes.

Samples	T_*o1*_	T_*p1*_	T_*c1*_	ΔH_1_	T_*o2*_	T_*p2*_	T_*c2*_	ΔH_2_	ΔH_*t*_
WS (50%)-GMP-100	88.6 ± 0.1a	97.5 ± 1.0b	101.9 ± 0.2abc	2.6 ± 0.1a	ND	ND	ND	ND	2.6 ± 0.1a
WS (60%)-GMP-100	89.5 ± 0.3ab	98.6 ± 0.3c	101.9 ± 0.8abc	3.3 ± 0.1b	ND	ND	ND	ND	3.3 ± 0.1b
WS (70%)-GMP-100	92.9 ± 0.6cd	98.1 ± 0.1bc	102.0 ± 0.3abc	3.5 ± 0.1b	ND	ND	ND	ND	3.5 ± 0.1b
WS (80%)-GMP-100	94.5 ± 0.5d	98.6 ± 0.3c	102.1 ± 0.3abc	4.5 ± 0.1c	ND	ND	ND	ND	4.5 ± 0.1c
WS (90%)-GMP-100	94.6 ± 0.3d	98.8 ± 0.1c	102.2 ± 0.3bc	5.3 ± 0.1d	ND	ND	ND	ND	5.3 ± 0.1d
WS (50%)-GMP-100-90	89.7 ± 0.0ab	95.7 ± 0.4a	102.7 ± 0.2c	3.3 ± 0.1b	ND	ND	ND	ND	3.3 ± 0.1b
WS (60%)-GMP-100-90	89.4 ± 0.1ab	96.1 ± 0.5a	102.5 ± 0.5c	4.6 ± 0.3c	111.4 ± 1.1a	119.2 ± 0.4d	121.4 ± 0.4abc	0.9 ± 0.0a	5.5 ± 0.2d
WS (70%)-GMP-100-90	89.4 ± 1.3ab	95.3 ± 0.5a	101.4 ± 0.5ab	4.3 ± 0.0c	111.6 ± 0.3ab	118.2 ± 0.2bcd	121.7 ± 0.7bc	1.7 ± 0.1d	6.0 ± 0.1e
WS (80%)-GMP-100-90	91.2 ± 3.4bc	95.4 ± 0.5a	101.0 ± 0.7a	3.3 ± 0.2b	111.5 ± 0.9a	118.8 ± 1.0cd	122.6 ± 1.3c	3.0 ± 0.0e	6.4 ± 0.3f
WS (90%)-GMP-100-90	89.1 ± 0.1ab	97.3 ± 1.5b	102.4 ± 1.3bc	3.3 ± 0.1b	112.6 ± 0.2bc	116.6 ± 0.2ab	120.3 ± 0.3ab	3.7 ± 0.1g	7.0 ± 0.1g

The different letters marked after the data indicate significant differences exist between the data (*p* < 0.05). T_*o*1_, T_*p*1_, T_*c*1_ (°C), ΔH_1_ (J/g) and T_*o*2_, T_*p*2_, T_*c*2_, ΔH_2_ are the onset temperature, peak temperature, conclusion temperature, and enthalpy change of type I complex and type II complex, respectively; ΔH_t_, total enthalpy change.

### 3.2 X-ray diffraction

**FIGURE 2:**
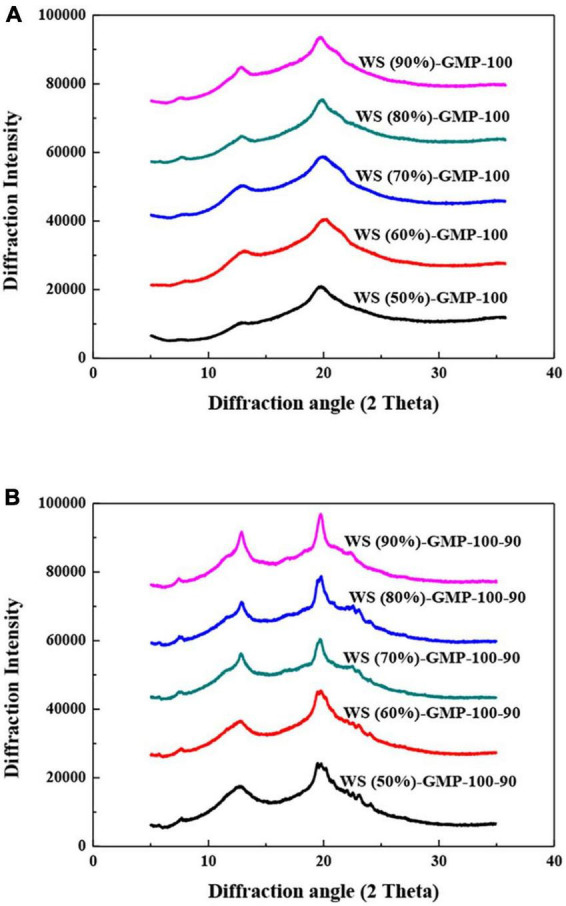
X-ray diffraction analysis (XRD) curves of WS-GMP complexes. **(A)** WS-GMP complexes were prepared at 100°C for 10 min. **(B)** WS-GMP complexes were prepared at 100°C for 10 min followed by complexing at 90°C for 60 min. WS (*n*%), wheat starch with *n*% water content; GMP, glycerol monopalmitin.

Two stronger peaks at 12.9 and 19.8° (2θ) and one weak peak at 7.5° (2θ), representing V-type crystallites were showed in the X-ray diffraction patterns of WS-GMP complexes (Figures 2A, B). The XRD peaks with broad and flat reflections of complexes were proposed to represent the formation of type I complexes, differently, type II complexes have the narrow and sharp V-type peaks (30, 31). The obvious broad and flat peaks at 12.9 and 19.8° (2θ) of WS-GMP-100 complexes indicated the formation of type I complexes. The changes of broad-and-blunt into sharp-and-narrow XRD peaks of WS-GMP-100-90 complexes with increasing the water content indicate the transformation of less stable type I complexes into stable type II complexes. These results are consistent with the DSC results. Compared to WS-GMP-100 complexes, the WS-GMP-100-90 complexes showed shaper V-type diffraction peaks, suggesting the formation of more ordered complexes after heating further at 90°C.

### 3.3 LCM-Raman spectroscopy

**FIGURE 3:**
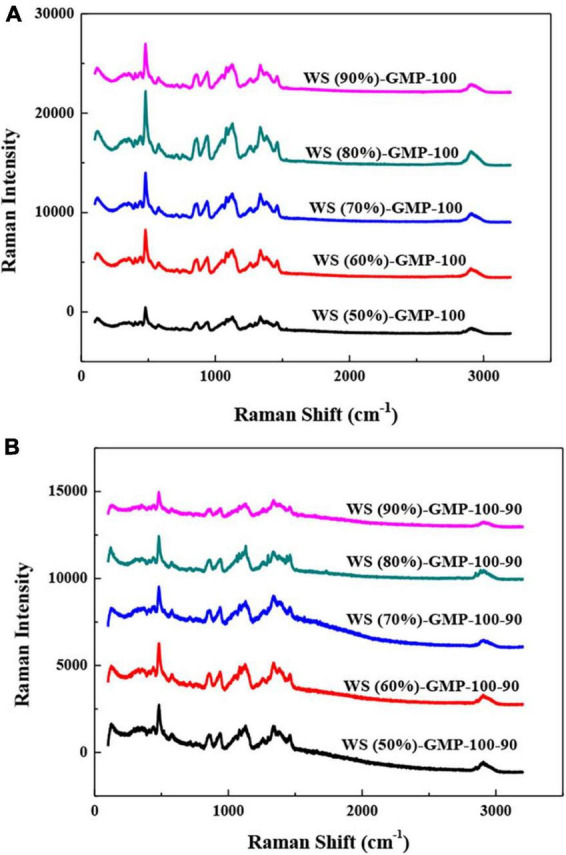
Laser confocal micro-Raman spectra of WS-GMP complexes. **(A)** WS-GMP complexes were prepared at 100°C for 10 min. **(B)** WS-GMP complexes were prepared at 100°C for 10 min followed by complexing at 90°C for 60 min. WS (*n*%), wheat starch with *n*% water content; GMP, glycerol monopalmitin.

The short-range order of WS-GMP complexes was characterized by Raman spectra (Figures 3A, B). The lower FWHM_480_ means the better short-range ordered structure, that is to say, the more amount of ordered structure due to the formation of complexes (14). The value FWHM_480_ decreased from 16.67 for WS (50%)-GMP-100 to 15.34 for WS (90%)-GMP-100, and from 15.69 for WS (50%)-GMP-100-90 to 14.29 for WS (90%)-GMP-100-90 (Table 2), suggesting that the short-range order of complexes increased with increasing water content, which is generally consistent with XRD and DSC results. The FWHM_480_ of the WS-GMP-100-90 complexes samples is lower than that of the corresponding WS-GMP-100 complexes samples, suggesting that the short-range order of the complexes further increased after heating at 90°C.

**TABLE 2:** FWHM_480_ of the Raman Band of WS-GMP complexes.

Samples	FWHM_480_
WS (50%)-GMP-100	16.67 ± 0.20g
WS (60%)-GMP-100	16.35 ± 0.24fg
WS (70%)-GMP-100	16.16 ± 0.17ef
WS (80%)-GMP-100	15.82 ± 0.14de
WS (90%)-GMP-100	15.34 ± 0.22c
WS (50%)-GMP-100-90	15.69 ± 0.15cd
WS (60%)-GMP-100-90	15.41 ± 0.08c
WS (70%)-GMP-100-90	14.78 ± 0.28b
WS (80%)-GMP-100-90	14.74 ± 0.45b
WS (90%)-GMP-100-90	14.29 ± 0.05a

The different letters marked after the data indicate significant differences exist between the data (p < 0.05).

### 3.4 *In vitro* digestibility of WS-GMP complexes

**FIGURE 4:**
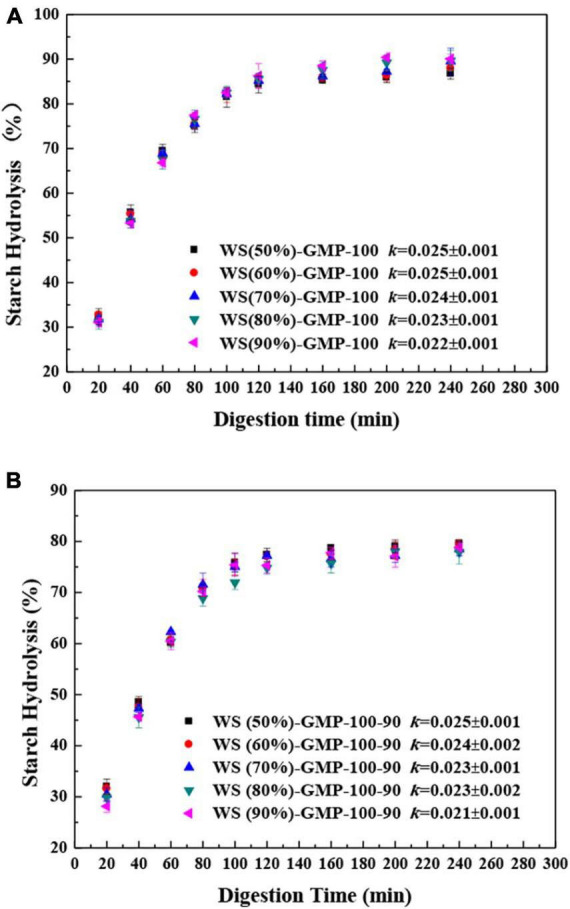
*In vitro* starch digestion of WS-GMP complexes. **(A)** WS-GMP complexes were prepared at 100°C for 10 min. **(B)** WS-GMP complexes were prepared at 100 °C for 10 min followed by complexing at 90°C for 60 min. WS (*n*%), wheat starch with *n*% water content; GMP, glycerol monopalmitin.

The digestograms of the WS-GMP complexes are presented in Figures 4A, B. All WS-GMP complexes reached a plateau after rapid digestion in the initial 80 min and the digestion percentage after 2 h was around 85 and 75%, respectively, for WS-GMP-100 and WS-GMP-100-90 complexes. The values of *k* decreased slightly with increasing water content for the WS-GMP complexes, and the *k*-value of the WS-GMP-100-90 complexes and the counterpart WS-GMP-100 complexes had little difference, for example, the *k*-values are 0.022 and 0.021 for the WS (90%)-GMP-100 complexes and WS (90%)-GMP-100-90 complexes, respectively. These results indicated that the final extent of starch amylolysis decreased after heating further at 90°C, but the digestion rate was little affected by the water content and the heating condition.

For further analysis of the digestibility of complexes, the RDS, SDS, and RS content were calculated (Table 3). They were little affected by the water content for the WS-GMP-100 complexes and WS-GMP-100-90 complexes. However, comparing with WS-GMP-100 complexes, WS-GMP-100-90 complex has lower RDS and SDS content and higher RS content. According to DSC and XRD results, the most obvious difference between the complexes obtained under two different processing conditions is the formation of type I and type II complexes with different structural stability. WS-GMP-100-90 complexes have more ordered type II complexes, resulting in obvious differences in RS, SDS, and RDS content between the WS-GMP-100 complexes and WS-GMP-100-90 complexes. The most obvious thing is that different amounts of complexes are obtained under the condition of different water content. Therefore, the formation of different crystal types complexes with different structural stability, rather than the number of complexes, have a more significant impact on the digestion of WS-GMP complexes.

**TABLE 3:** Starch hydrolysis fractions of WS-GMP complexes.

Samples	RDS	SDS	RS
WS (50%)-GMP-100	32.3 ± 1.9c	52.0 ± 1.4b	14.7 ± 0.8a
WS (60%)-GMP-100	32.7 ± 0.7c	52.2 ± 1.8b	15.0 ± 0.6a
WS (70%)-GMP-100	31.8 ± 0.7bc	53.4 ± 1.5bc	14.8 ± 0.8a
WS (80%)-GMP-100	31.1 ± 1.6bc	54.4 ± 1.4bc	14.5 ± 0.8a
WS (90%)-GMP-100	31.1 ± 1.1bc	55.2 ± 1.4c	15.3 ± 0.7a
WS (50%)-GMP-100-90	32.0 ± 1.5bc	45.4 ± 1.9a	22.6 ± 0.8b
WS (60%)-GMP-100-90	31.6 ± 1.0bc	45.4 ± 0.9a	23.0 ± 1.0b
WS (70%)-GMP-100-90	30.5 ± 1.3bc	46.7 ± 1.6a	22.8 ± 0.3b
WS (80%)-GMP-100-90	29.8 ± 0.8ab	45.0 ± 1.5a	25.2 ± 0.9c
WS (90%)-GMP-100-90	28.1 ± 0.8a	47.2 ± 1.6a	24.7 ± 0.6c

The different letters marked after the data indicate significant differences exist between the data (p < 0.05).

### 3.5 The correlation between the structures and digestibility of complexes

Many studies have demonstrated that the formation and structure of starch-lipid complex are important factors in determining the resistance of starch to enzymatic hydrolysis (32, 33). In general, complexes with the greater structural order have the lower digestibility. In order to further analyze the relationships between the structures of WS-GMP complexes and *in vitro* starch digestibility, Pearson correlation analyses were performed (Table 4). Results showed that the total enthalpy changes (ΔH_*t*_) of WS-GMP complexes were negatively correlated with the RDS (*r* = −0.883, *p* < 0.01) and *k*-value (*r* = −0.845, *p* < 0.01), and positively correlated with the RS (*r* = 0.700, *p* < 0.05). Previous studies observed a strong positive correlation between enthalpy and RS contents (32). The FWHM_480_ of WS-GMP complexes positively correlated with the RDS (*r* = 0.900, *p* < 0.01) and *k* value (*r* = 0.800, *p* < 0.01), but negatively correlated with the RS (*r* = −0.807, *p* < 0.01). Compared with the correlation coefficient between RS/RDS and ΔH_*t*_, RS/RDS had stronger correlation with FWHM_480_. This result indicated that short-range molecular order plays a more significant role in the content of RS and RDS. Similar results of a stronger positive correlation between the short-range ordering and RS contents were found in previous studies (32, 34). The above results of correlation analyses also suggested that the decreased digestion of WS-GMP complexes is more related to the increasing ordered structure.

**TABLE 4:** Pearson’s correlation analysis between ΔH_*t*_, FWHM_480_ and digestion parameters of WS-GMP complexes.

	ΔH_*t*_	FWHM_480_
RDS	-0.883**	0.900**
SDS	-0.455	0.574
RS	0.700*	-0.807**
*K*	-0.845**	0.800**

* and **Correlation is significant at the 0.05 level and 0.01 level, respectively.

## 4 Conclusion

The water content and heating conditions can affect the formation of starch-lipid complexes. The higher water content and the heating conditions of 90°C for 60 min after 100°C for 10 min are helpful for the formation of more complexes with the better short-range order. The heating conditions of 90°C for 60 min after 100°C for 10 min facilitates the formation of type II complexes, and the amounts of type II complexes increased with increasing water content. The digestion rate of WS-GMP complexes decreased slightly with increasing water content, however, the extent of starch digestion decreased significantly after further heating at 90°C for 60 min. The digestion difference of WS-GMP complexes are mainly determined by the structure order caused by different heating conditions rather than the number of complexes. This study provided new insights into the formation of starch-lipid complexes, which is of great significance for modulation of the functional and nutritive properties of starchy food.

## Data availability statement

The original contributions presented in the study are included in the article/supplementary material, further inquiries can be directed to the corresponding author.

## Author contributions

FJ: Data curation, Formal analysis, Investigation, Writing – original draft. ZL: Data curation, Formal analysis, Investigation, Writing – original draft. JN: Writing – review and editing. JY: Writing – review and editing. SW: Validation, Writing – review and editing. XL: Conceptualization, Formal analysis, Funding acquisition, Supervision, Writing – review and editing.
